# MALDI-TOF High Mass Calibration up to 200 kDa Using Human Recombinant 16 kDa Protein Histidine Phosphatase Aggregates

**DOI:** 10.1371/journal.pone.0023612

**Published:** 2011-08-18

**Authors:** Katrin Ludwig, Schähdi Habbach, Josef Krieglstein, Susanne Klumpp, Simone König

**Affiliations:** 1 Integrated Functional Genomics, Core Unit of the Interdisciplinary Center for Clinical Research, Medical Faculty, University of Münster, Münster, Germany; 2 Institute for Pharmaceutical and Medical Chemistry, University of Münster, Münster, Germany; California Institute of Technology, United States of America

## Abstract

**Background:**

Protein histidine phosphatase (PHP) is an enzyme which removes phosphate groups from histidine residues. It was described for vertebrates in the year 2002. The recombinant human 16 kDa protein forms multimeric complexes in physiological buffer and in the gas phase. High-mass calibration in matrix-assisted laser desorption/ionization time-of-flight mass spectrometry (MALDI-TOF MS) has remained a problem due to the lack of suitable standards. Large proteins can hardly be freed of their substructural microheterogeneity by classical purification procedures so that their use as calibrants is limited. A small adduct-forming protein of validated quality is a valuable alternative for that purpose.

**Methodology/Principal Findings:**

Three major PHP clusters of ∼113, 209 and >600 kDa were observed in gel filtration analysis. Re-chromatography of the monomer peak showed the same cluster distribution. The tendency to associate was detected also in MALDI-TOF MS measuring regular adducts up to 200 kDa.

**Conclusions/Significance:**

PHP forms multimers consisting of up to more than 35 protein molecules. In MALDI-TOF MS it generates adduct ions every 16 kDa. The protein can be produced with high quality so that its use as calibration compound for high mass ranges above 100 kDa, where standards are difficult to obtain, is feasible.

## Introduction

Protein histidine phosphatase (PHP) hydrolytically cleaves phosphate groups from histidine residues. It was described for vertebrates in the year 2002 [1], [2]. ATP-citrate lyase (ACL) has been identified as a physiological substrate [3]; a second is the β-subunit of heterotrimer G-protein [4]. In 2008, dephosphorylation of potassium channel KCa3.1 was shown, a protein whose phosphorylation by nucleoside diphosphate kinase B is required for activation of KCa3.1 and CD4 T cells [5]. More proteins such as glutathione-S transferase and hemoglobin have been found to interact with PHP [6] and further functional studies are ongoing to characterize PHP.

Recombinantly expressed human 16 kDa PHP [7] forms multimeric complexes in physiological buffer as is described in this work. Regular series of PHP multimers can also be observed up to 200 kDa in matrix-assisted laser desorption/ionization time-of-flight mass spectrometry (MALDI-TOF MS). Since high-quality PHP can be produced, its use as calibration compound for high mass ranges is discussed.

Available calibration standards typically cover the range up to 100 kDa and are based on well known proteins such as apomyoglobin, trypsinogen and bovine serum albumin (BSA) of high to moderate purity. While proteins of larger molecular weight can be purchased, e.g. standards for gel electrophoresis, they are often mircoheterogenous and their purity is mostly not sufficient for MS measurement resulting in peaks of low resolution. Authors have suggested the use of different classes of aggregate or macromolecule forming substances such as poly(dimethylsiloxanes) [8], but those compounds are only available as mixtures of average molecular weight so that their use as calibrants is limited to very specific applications. A small adduct-forming protein of validated quality such as PHP may prove to be a valuable alternative for that purpose.

## Results and Discussion

**Figure 1:**
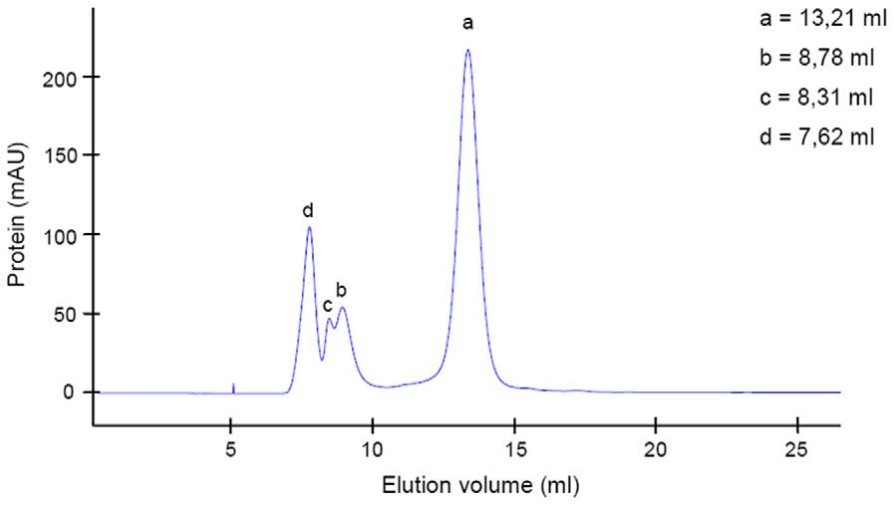
PHP gel filtration profile. (Superdex 75, 4°C, 280 nm). a) Monomer. Fractions b) and c) were pooled for further processing (see Figure 2).

**Figure 2:**
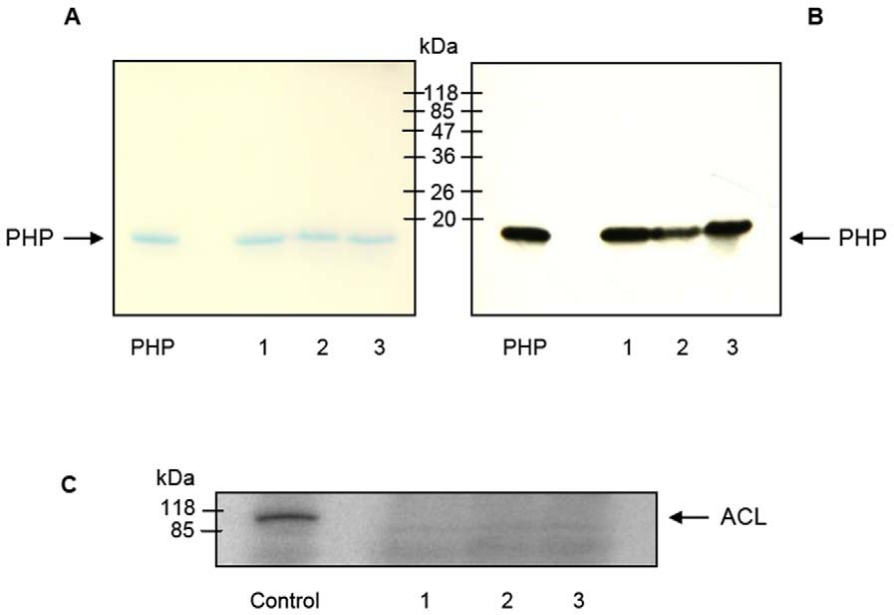
Quality control of gel chromatography fractions. (Figure 1; a) sample 1, b/c) sample 2, d) sample 3) with respect to PHP **C**) activity (dephosphorylation of ACL) and **A/B**) purity. 1D-PAGE using PHP as control. **A**) Coomassie-staining. **B**) Detection with polyclonal PHP antibody.

First indications of PHP aggregation were observed in gel chromatography, a method which is often employed to separate protein mixtures. Gel filtration analysis using Superdex 75 showed four peaks (Figure 1). While the most intense signal could be assigned to PHP monomer, the other peaks were outside the separation range of the selected column indicating the presence of much larger protein forms. Neither contamination nor cleavage of PHP could be detected in these fractions as was demonstrated by one-dimensional polyacrylamide gel electrophoresis 1D-PAGE (Figure 2A). Polyclonal PHP antibody recognized the protein in all fractions (Figure 2B) and a test for PHP activity confirmed that all fractions dephosphorylated ACL (Figure 2C). The tendency of PHP to aggregate increased with storage time (1 day to 3 months, Figure S1) and experiment temperature (4°C *versus* room temperature; Figure S2). Aggregates formed independently of protein concentration (tested for 10, 100, 1000 µg / 100 µl; Figure S3). An active mutant missing eight amino acids at the N-terminus [9] also aggregated (Figure S4). When gel chromatography fractions were re-chromatographed, they eluted with a similar profile as the original solution (Figure S5) indicating that equilibrium formed among the different types of aggregates. Collection of only one type of aggregate was therefore not possible. It could also not yet be determined whether PHP activity was due to the monomer or one of the aggregate forms. The presence of PHP in each re-chromatographed fraction was proven by Western blot analysis (Figure S6).

**Figure 3:**
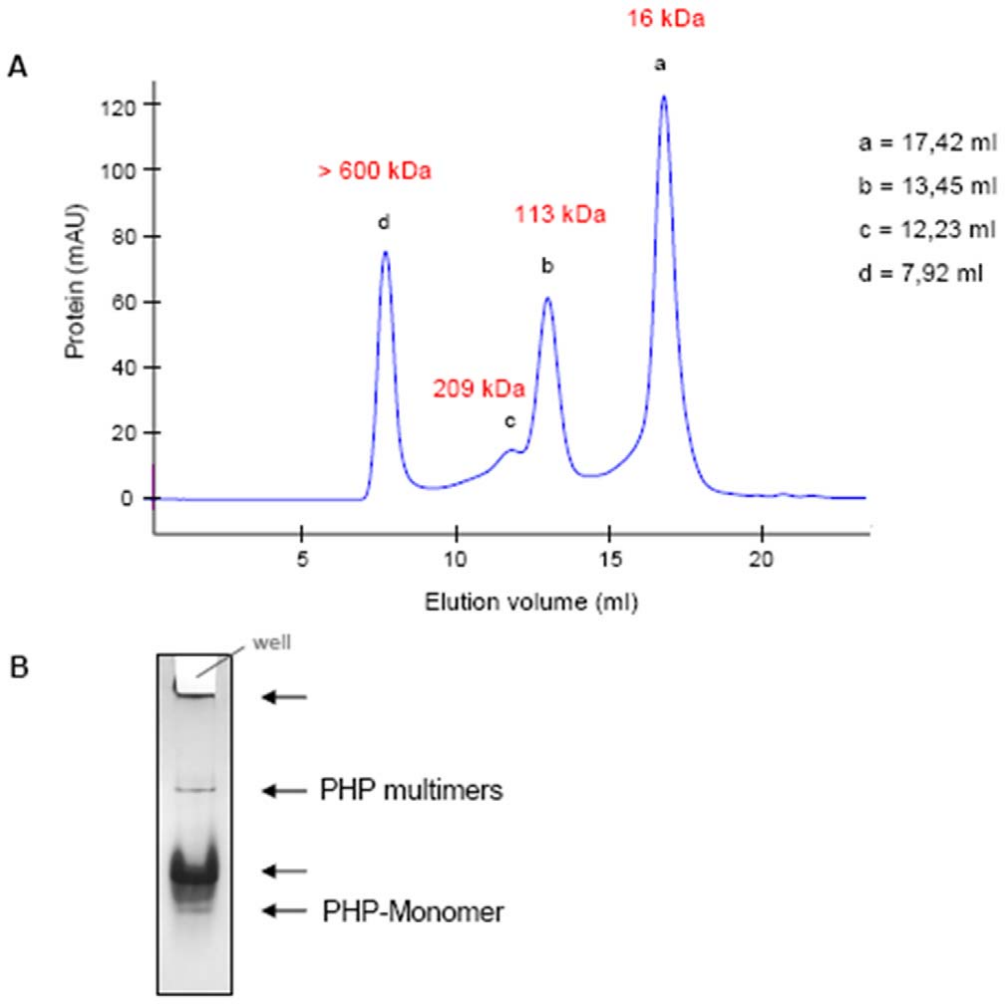
Determination of PHP aggregate sizes. Using **A**) Superdex 200 (Signal d was outside the separation range of the column so that cluster size was estimated.) and **B**) native gel electrophoresis (several multimer signals, but only one monomer band).

For determination of aggregate sizes, a gel filtration column for larger mass ranges was employed (Superdex 200, separation range 10–600 kDa). Cluster sizes of ∼113 kDa, 209 Da and>600 kDa were measured indicating multimers of 7, 13, and more than 37 units, respectively (Figure 3A). Separation of the same sample on native gel electrophoresis showed one monomer band and three multimer bands as well as PHP material, which did not enter the gel and likely resulted from higher aggregates (Figure 3B). Since no marker substance was available, molecular weights of the bands could only be estimated using the gel filtration profile, but it was striking that the monomer did not seem to be the most abundant species. When gel filtration analysis was performed using denaturing conditions (Figure S7), the monomer was absent and only one type of very large aggregate was observed (∼500 kDa); these clusters eluted as a single peak. This represents an interesting phenomenon, because in denaturing PAGE the monomer was observed at the expected mass. Obviously, individual methodological aspects such as the application of electric field strength in PAGE play a role, but these parameters were not further elucidated. Although the functional properties of PHP are impaired after denaturing, the protocol is potentially useful for the separation of PHP from other proteins in cases where activity is not important.

**Figure 4:**
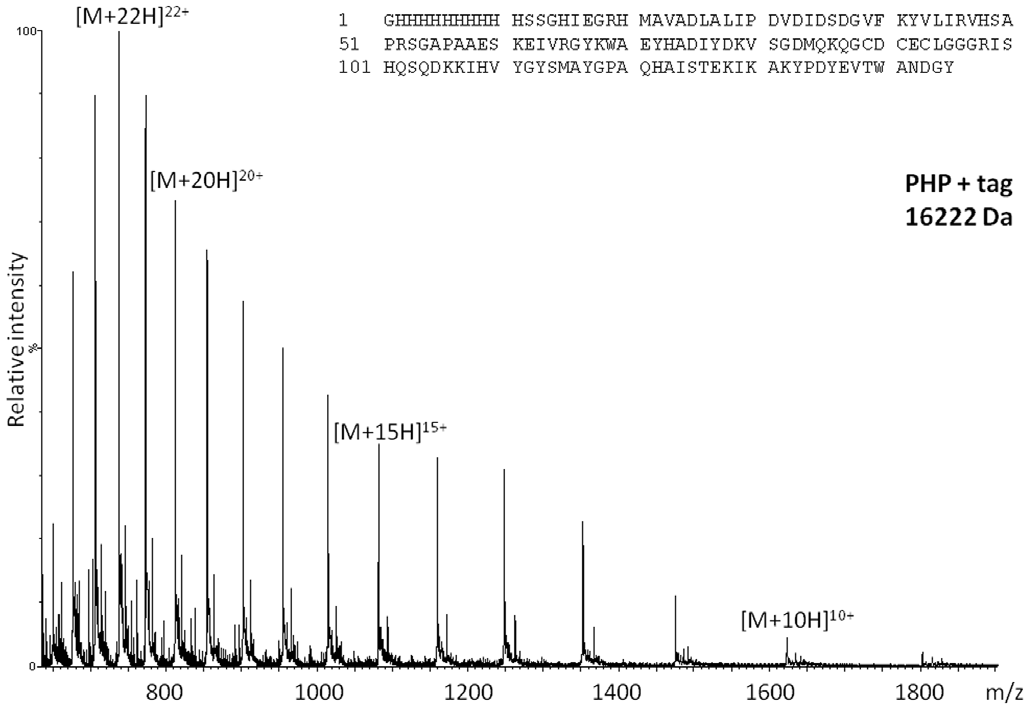
Q-TOF nanospray mass spectrum of desalted PHP. Satellite peaks correspond to residual contaminants from protein isolation, which cannot be easily removed. PHP sequence is given as inset.

**Figure 5:**
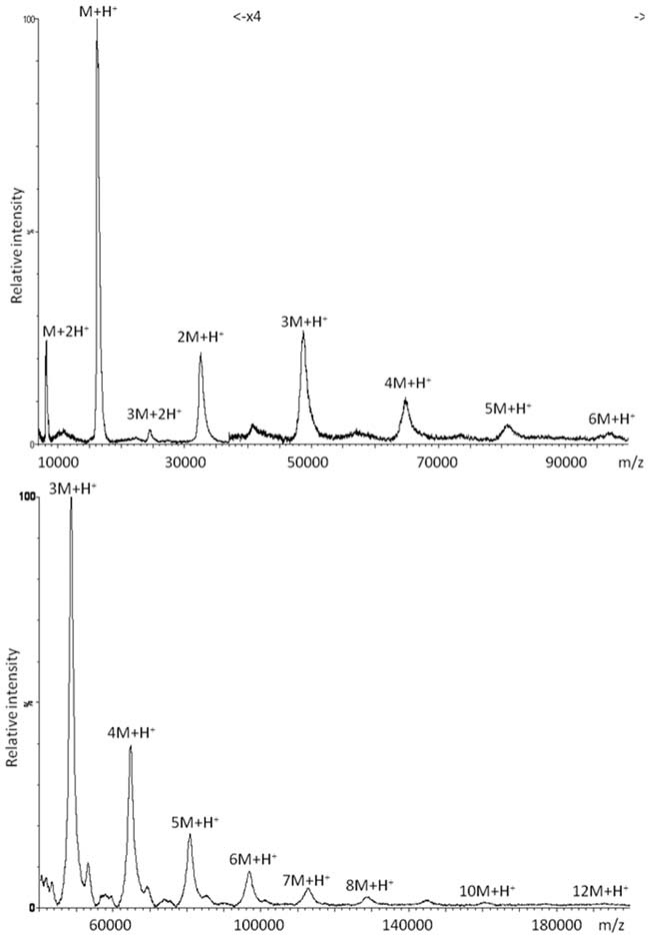
MALDI-TOF spectra of His-tagged PHP. Top: Monomer up to hexamer (observe zoom for range >*m/z* 3700); Below: Trimer to dodecamer. For peak assignment see Table 1.

PHP was expressed with His-tag having a molecular weight of 16222 Da. It could be produced with >90 % purity as estimated by electrospray ionization (ESI)-MS measurement (Figure 4). When PHP was investigated with MALDI-TOF MS using typical protein preparations with sinapinic acid (SA), adduct ions were observed with decreasing intensity to higher mass ranges (Figure 5). This was also true for PHP without His-tag (Figure S8). There were no favored ion species with particularly high intensity (magic numbers). Adduct formation was regular and reached up to *m/z* values of ∼200 kDa, a feature which can be used for calibration in regions where mass standards are scarce. PHP delivers a calibration point every 16 kDa (Table 1) and some of the doubly-charged ion species can be used as well. Since PHP reliably delivers a large range of well resolved adduct ions, it is a valuable alternative to high mass calibration standards of lower purity. There is also potential for the visualization of even larger clusters by adapting the protocol with respect to matrix and sample composition or the use of instruments optimized for high mass ions.

**Table 1:** Average molecular weights of singly and doubly protonated PHP multimers.

n	M+H^+^	M+2H^+^
1	16223	8112
2	32445	16223
3	48667	24334
4	64889	32445
5	81111	40556
6	97333	48667
7	113555	56778
8	129777	64889
9	145999	73000
10	162221	81111
11	178443	89222
12	194665	97333

## Materials and Methods

PHP was recombinantly expressed as described earlier [7]. Gel filtration was performed using Superdex 75 or Superdex 200 and Äkta (Amersham/GE) at 4°C. PHP was applied at 1 mg in 100 µl. Running buffer was 20 mM Tris/HCl (pH 7.5), 100 mM NaCl. Elution profiles were measured at 280 nm. The marker BSA eluted from this column at 9 ml so that only the size of the PHP monomer could be confirmed. Fractions from gel filtration were concentrated using ultracentrifugal filter devices (Amicon, Millipore).

For 1D-PAGE, 5 µl sample buffer (130 mM Tris/HCl, pH 6.8; 10 % SDS, 10 % mercaptoethanol (ME), 20 % glycerol, 0.06 % bromophenylblue) was added. The sample was heated to 95°C for 5 min and subjected to SDS-PAGE (15 %; 2 µg protein per well). Coomassie staining was used. Native gel electrophoresis was performed with 9 µg PHP. The sample was combined 1∶1 v/v with sample buffer and applied to the native 15 % gel. Silver staining was used for detection. For immunological detection of PHP, 500 ng protein was applied per well. It was carried out using polyclonal PHP-antibody (described in ref. [1], 1∶400, 0.1 % BSA) and bands were visualized with X-ray film (15 s).

For dephosphorylation of ACL, 100 µg rabbit liver extract (group of J. Krieglstein at the Institute for Pharmacology and Toxicology of the University of Marburg; All animal work was conducted according to national guidelines. [10]) was incubated in the presence of 2 µCi [γ-^32^P]ATP with 1 µM ATP, 25 mM Tris/HCl (pH 7.5) and 5 mM EDTA for 15 min at 37°C. To test for activity, 600 ng PHP (in 25 mM Tris/HCl, pH 7.5; 5 mM EDTA) was added (30 min, 37°C). Water was used for the control experiment. Samples were not heated before separation. For detection, an X-ray film was placed on top of the gel for 24 h.

The protein was desalted for MS using ZipTips C_18_ (Millipore, Bedford, USA). The tips were washed with elution buffer (75 % acetonitrile, 0.1 % trifluoroacetic acid (TFA)) and equilibrated with aqueous solvent (5 % methanol, 0.1 % TFA). Aqueous PHP solution (3.5 µg in 15 µl) was applied and rinsed with equilibration solution. The protein was eluted into 5 µl elution buffer. SA was used as matrix for MALDI-TOF MS. Following target preparation with 0.5 µl of saturated SA in acetone, 0.5 µl of PHP solution and 0.5 µl of saturated solution of SA in acetonitrile / 0.1 % TFA 1∶2 were added. Chemicals and solvents for MS (Sigma) were of highest quality. MALDImicroMX (Waters Corp., Manchester, UK) was used in positive linear mode with high mass detector turned on for measurements in mass ranges >50 kDa. For ESI-MS, Q-TOF Premier (Waters Corp.) was employed using a home-made nanospray source [11].

### Supporting Information

Supporting information providing additional data is made available.

## Supporting Information

Figure S1 **Dependence of aggregate formation on storage time.** Gel filtration elution profiles for PHP stored at −80°C. PHP of a single preparation batch was stored for **A**) 1 day, **B**) 3 days or **C**) 3 months.
(TIF)

Figure S2 **Temperature dependence of PHP aggregation.** Elution profiles of PHP at **A**) 4°C and **B**) room temperature.
(TIF)

Figure S3 **Dependence of aggregate formation on protein concentration.** Elution profiles of PHP when **A**) 1000 µg, **B**) 100 µg and **C**) 10 µg of the same batch were separated on Superdex 200 (100 µl application volume; 20 mM Tris/HCl, pH 7.5, 100 mM NaCl, 4°C).
(TIF)

Figure S4 **Dependence of PHP aggregation on N-terminal amino acids.** Elution profile of PHP mutant with N-terminal deletion of eight amino acids (NΔ8-PHP [11]).
(TIF)

Figure S5 **Re-chromatography of PHP fractions.** **A**) Elution profile of PHP using Superdex 75 column. Peak a^1^ was collected; peaks b^1^-d^1^ were pooled. **B**) Elution profile of fraction a^1^. **C**) Elution profile of pool b^1^, c^1^ and d^1^. See Figure S6.
(TIF)

Figure S6 **Western blot analysis of gel chromatography peak fractions.** Polyclonal PHP-antibody was used. Fraction a^2^ (sample 1), b^2^ (2) and c^2^ (3) shown in Figure S5b and a ^3^ (4), b^3^ and c^3^ (5) and d^3^ from Figure S5c (6) were collected, concentrated (Amicon) and gel electrophoretically separated. (500 ng protein in 5 µl sample buffer, heated to 95°C for 5 min, 15 % SDS-PAGE).
(TIF)

Figure S7 **Influence of denaturing conditions on gel filtration analysis.** PHP was dissolved in 20 mM Tris/HCl, pH 7.5, 100 mM NaCl (1 mg PHP in 100 µl application volume, room temperature, Superdex 75) **A**) 2 % SDS and 0.1% 2-ME or **B**) 2 % SDS, 0.1 % 2-ME and 8 M urea, heated to 95°C for 10 min.
(TIF)

Figure S8 **Q-TOF nanospray mass spectrum of desalted tag-free PHP.** Satellite peaks correspond to residual contaminants from protein isolation, which are difficult to remove. The sequence is given as inset.
(TIF)
