# Supplementary figures and images for: MALDI-TOF High Mass Calibration up to 200 kDa Using Human Recombinant 16 kDa Protein Histidine Phosphatase Aggregates
Source: PLoS One. 2011 Aug 18;6(8):e23612. doi: 10.1371/journal.pone.0023612 (PMC3158095; doi:10.1371/journal.pone.0023612)

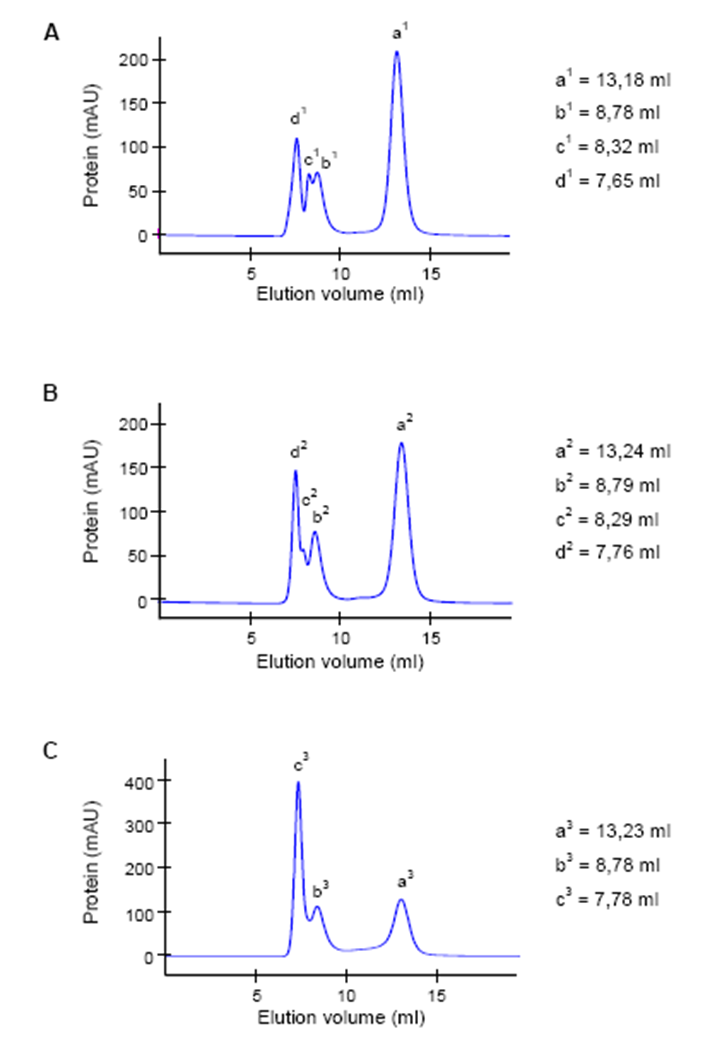

Supplement: Figure S1 — Dependence of aggregate formation on storage time. Gel filtration elution profiles for PHP stored at −80°C. PHP of a single preparation batch was stored for A) 1 day, B) 3 days or C) 3 months. (TIF) [file pone.0023612.s001.tif]

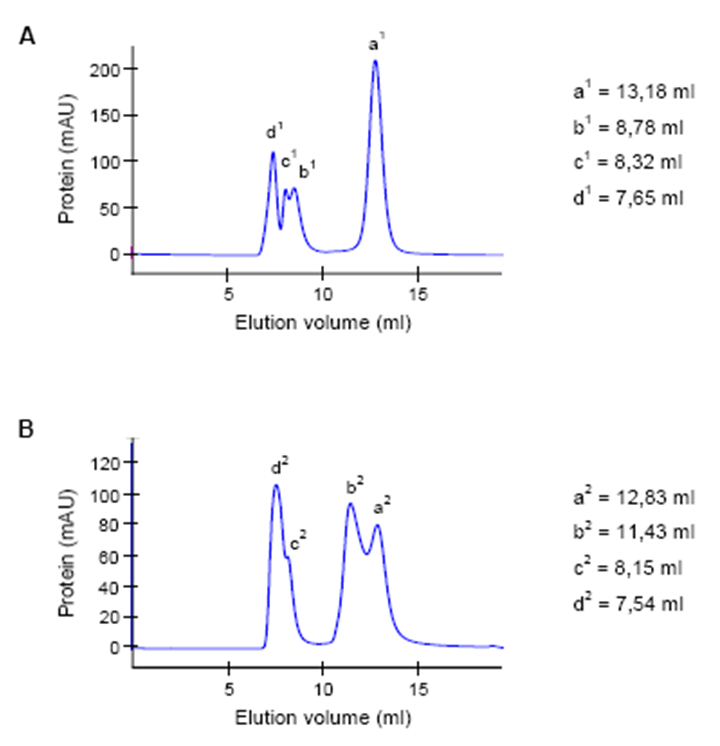

Supplement: Figure S2 — Temperature dependence of PHP aggregation. Elution profiles of PHP at A) 4°C and B) room temperature. (TIF) [file pone.0023612.s002.tif]

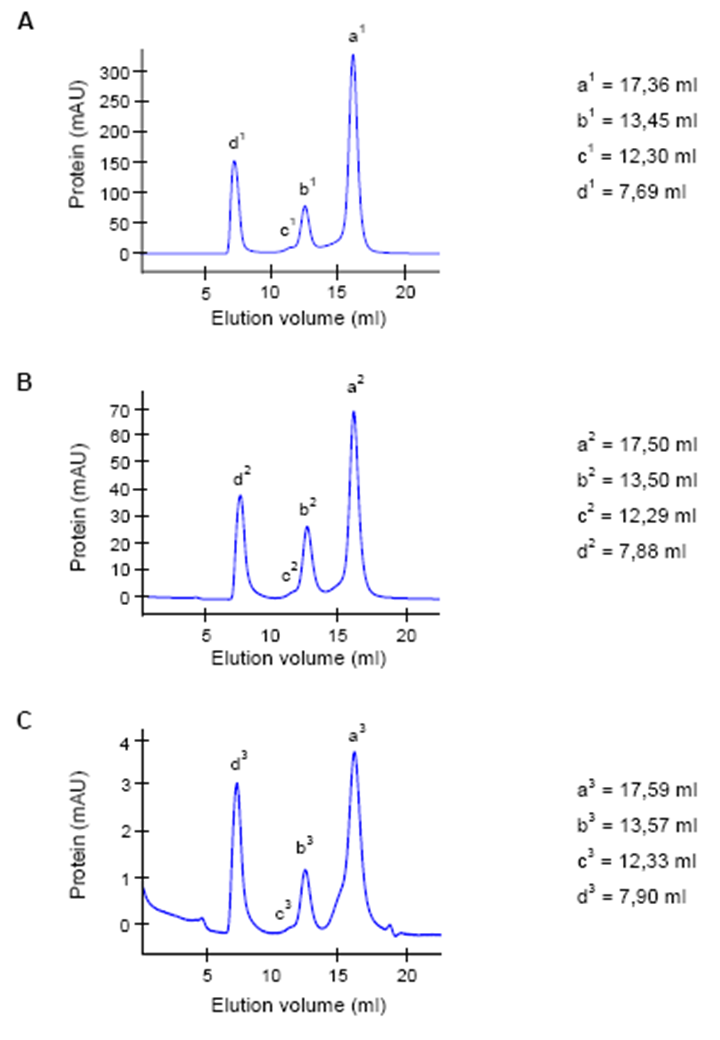

Supplement: Figure S3 — Dependence of aggregate formation on protein concentration. Elution profiles of PHP when A) 1000 µg, B) 100 µg and C) 10 µg of the same batch were separated on Superdex 200 (100 µl application volume; 20 mM Tris/HCl, pH 7.5, 100 mM NaCl, 4°C). (TIF) [file pone.0023612.s003.tif]

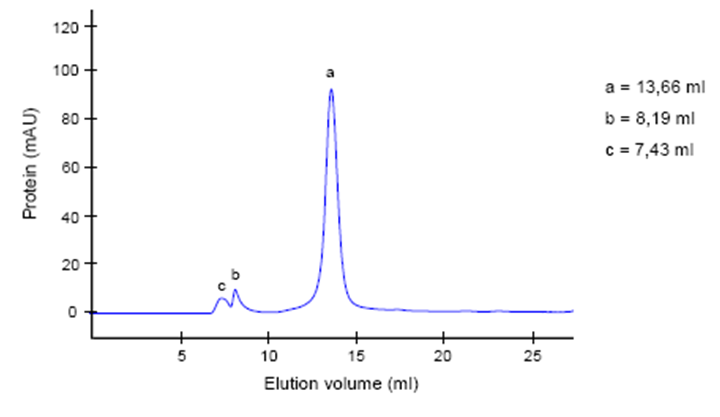

Supplement: Figure S4 — Dependence of PHP aggregation on N-terminal amino acids. Elution profile of PHP mutant with N-terminal deletion of eight amino acids (NΔ8-PHP [11]). (TIF) [file pone.0023612.s004.tif]

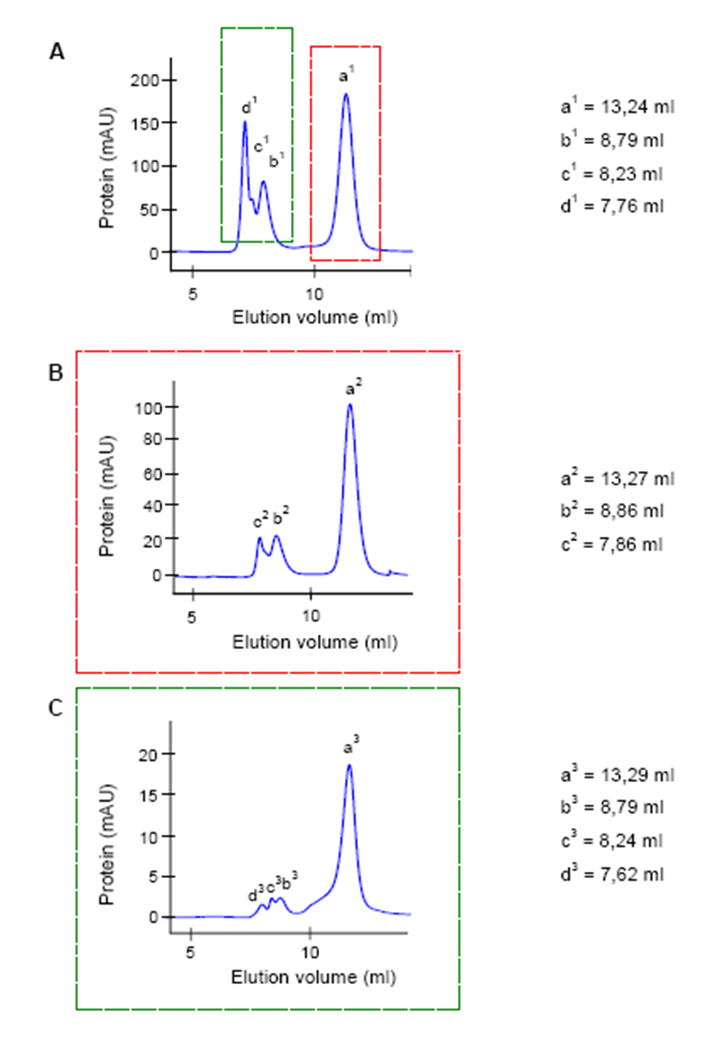

Supplement: Figure S5 — Re-chromatography of PHP fractions. A) Elution profile of PHP using Superdex 75 column. Peak a1 was collected; peaks b1-d1 were pooled. B) Elution profile of fraction a1. C) Elution profile of pool b1, c1 and d1. See Figure S6. (TIF) [file pone.0023612.s005.tif]

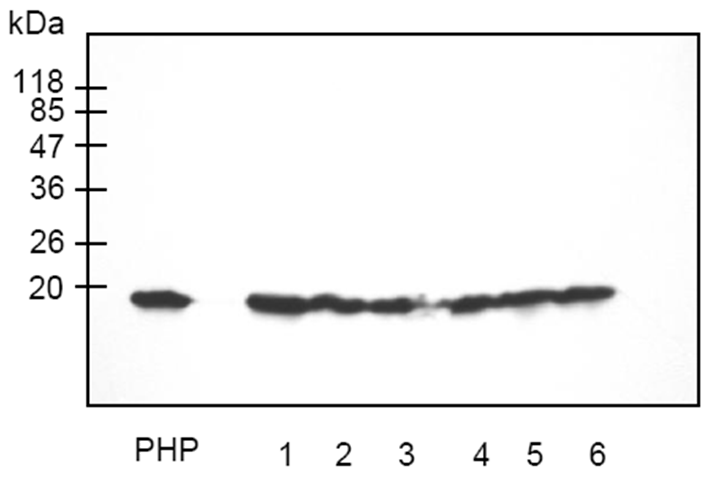

Supplement: Figure S6 — Western blot analysis of gel chromatography peak fractions. Polyclonal PHP-antibody was used. Fraction a2 (sample 1), b2 (2) and c2 (3) shown in Figure S5b and a 3 (4), b3 and c3 (5) and d3 from Figure S5c (6) were collected, concentrated (Amicon) and gel electrophoretically separated. (500 ng protein in 5 µl sample buffer, heated to 95°C for 5 min, 15 % SDS-PAGE). (TIF) [file pone.0023612.s006.tif]

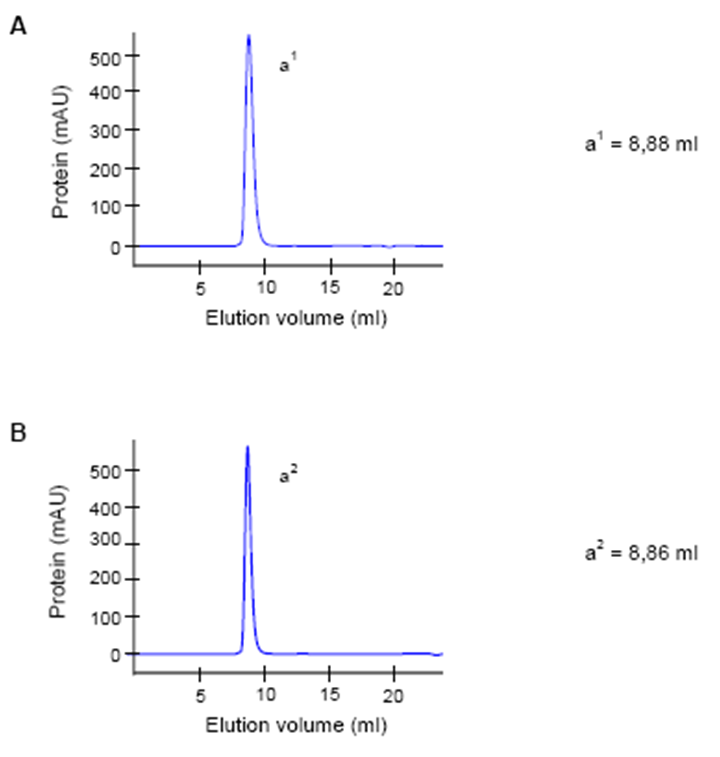

Supplement: Figure S7 — Influence of denaturing conditions on gel filtration analysis. PHP was dissolved in 20 mM Tris/HCl, pH 7.5, 100 mM NaCl (1 mg PHP in 100 µl application volume, room temperature, Superdex 75) A) 2 % SDS and 0.1% 2-ME or B) 2 % SDS, 0.1 % 2-ME and 8 M urea, heated to 95°C for 10 min. (TIF) [file pone.0023612.s007.tif]

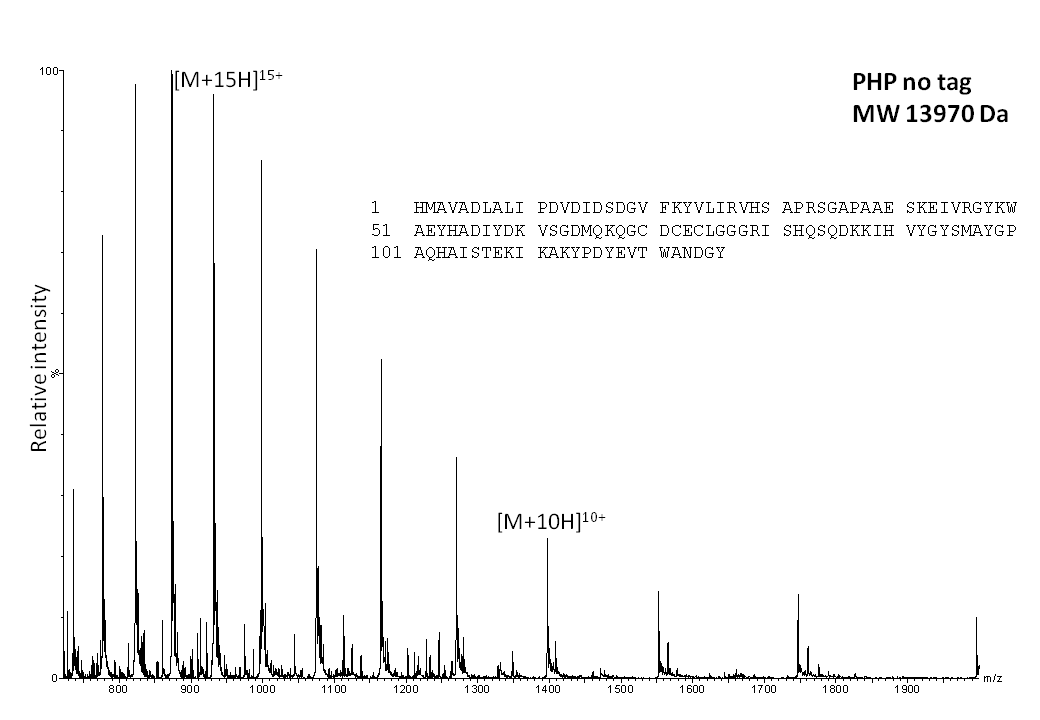

Supplement: Figure S8 — Q-TOF nanospray mass spectrum of desalted tag-free PHP. Satellite peaks correspond to residual contaminants from protein isolation, which are difficult to remove. The sequence is given as inset. (TIF) [file pone.0023612.s008.tif]
